# Supplementary material for: Retrospective Case-Control Study of 2017 G2P[4] Rotavirus Epidemic in Rural and Remote Australia
Source: Pathogens. 2020 Sep 26;9(10):790. doi: 10.3390/pathogens9100790 (PMC7601783; doi:10.3390/pathogens9100790)
Supplement: Supplementary file 1 [file pathogens-09-00790-s001.pdf]

pathogens
MDPI

Supplementary Materials:

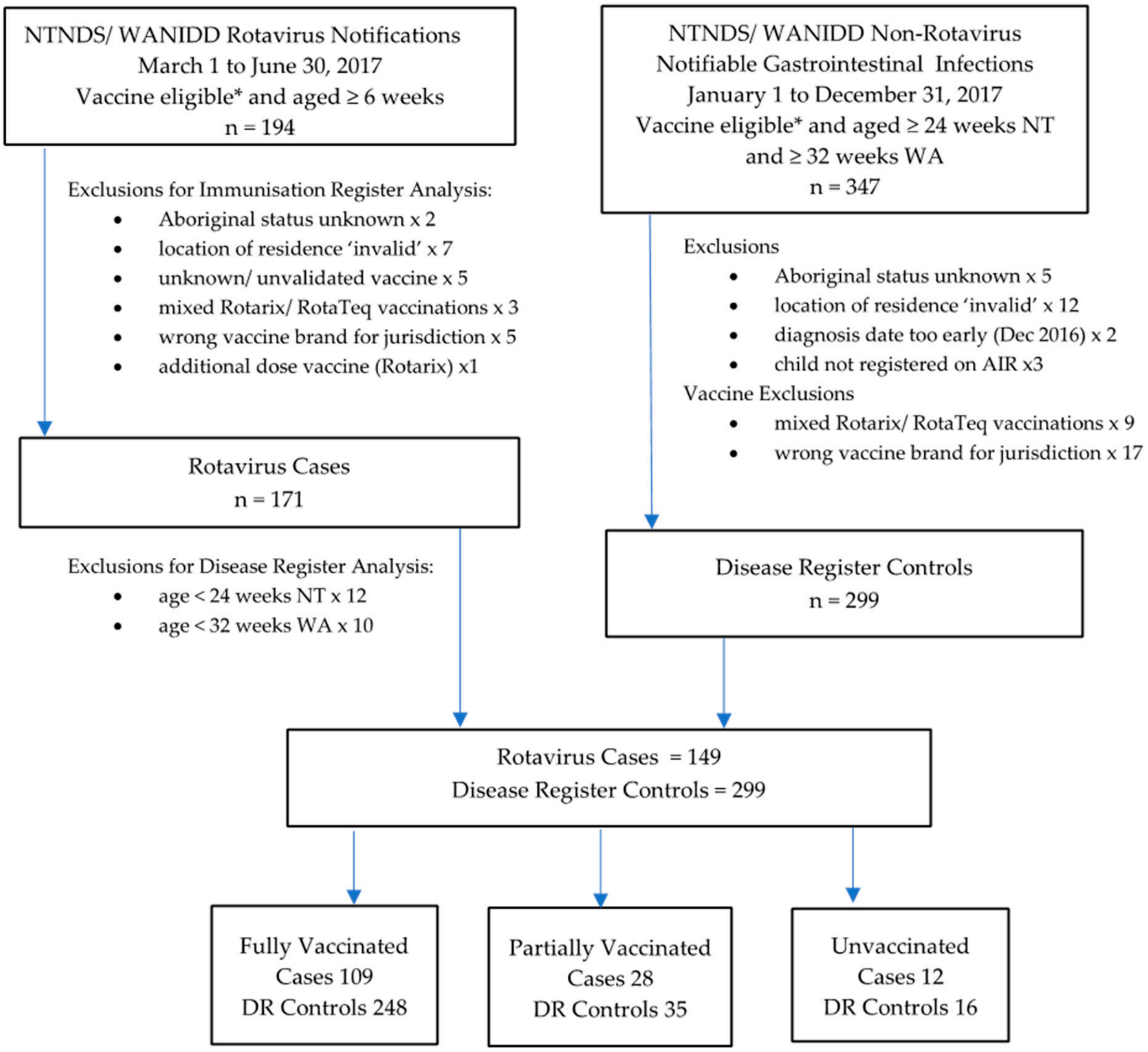

Figure S1. Selection of rotavirus cases and un-matched disease register controls for disease register nested case-control study.

*Vaccine Eligible: children eligible by date-of-birth to have received at least one dose of Rotarix vaccine (those born after July 1, 2006 in the Northern Territory) or at least one dose of RotaTeq vaccine (those born after 1 May 2009 in Western Australia).

Table S1. Baseline characteristics of rotavirus cases and disease register controls for the unmatched disease-register nested case-control study.

Characteristic	Rotavirus Cases		Disease Register Controls	
	NT	WA	NT	WA
	n = 71	n = 78	n = 123	n = 176
**Age**				
Median Age (months)	20	20	23	33
Age Range (months)	5 to 72	7 to 94	5 to 132	8 to 99
24 (NT)/ 32 (WA) wks to < 1 year	13 (18%)	13 (17%)	20 (16%)	17 (10%)
1 year to < 2 years	36 (51%)	34 (44%)	42 (34%)	44 (25%)
2 year to < 3 years	11 (16%)	15 (19%)	19 (15%)	38 (22%)
3 year to < 4 years	7 (10%)	5 (6%)	19 (15%)	23 (13%)
4 years to < 5 years	3 (4%)	5 (6%)	5 (4%)	18 (10%)
≥ 5 years	1 (1%)	6 (8%)	18 (15%)	36 (20%)
**Sex**				
Female	35 (49%)	39 (50%)	58 (47%)	85 (48%)
Male	36 (51%)	39 (50%)	65 (53%)	91 (52%)
**Aboriginal Status**				
Aboriginal	59 (83%)	58 (74%)	110 (89%)	73 (41%)
Non-Aboriginal	12 (17%)	20 (26%)	13 (11%)	103 (59%)
**Geographical Area**				
Alice Springs	10 (14%)		92 (75%)	
Barkly	61 (86%)		31 (25%)	
Goldfields		14 (18%)		48 (27%)
Kimberley		47 (60%)		61 (35%)
Pilbara		17 (22%)		67 (38%)
**Organism**				
Rotavirus	71 (100%)	78 (100%)		
Rotavirus G2P[4]	36 (51%)	54 (69%)		
Campylobacter			22 (18%)	33 (19%)
Cryptosporidium			14 (11%)	27 (15%)
Salmonella			20 (16%)	80 (46%)
Shigella			67 (55%)	36 (20%)
**Hospitalisation**				
Yes	56 (79%)	30 (39%)	67 (54%)	38 (22%)
No	15 (21%)	37 (47%)	49 (40%)	97 (55%)
Unknown	0 (0%)	11 (14%)	7 (6%)	41 (23%)
**Vaccination**				
0 doses	3 (4%)	9 (12%)	9 (7%)	7 (4%)
1 doses	10 (14%)	2 (2%)	6 (5%)	7 (4%)
2 doses	58 (82%)	16 (21%)	108 (88%)	22 (12%)
3 doses		51 (65%)		140 (80%)

Table S2. Odds Ratio of vaccination in rotavirus cases versus controls in the matched population-based analysis and the disease register nested analysis (full results).

	Population-Based Analysis			Disease Register Analysis		
**Immunisation Status**	**Cases**	**Controls**	**Odds Ratio (95% CI)**	**Cases**	**Controls**	**Odds Ratio (95% CI)**
**Any Dose vs None**	n = 171	n = 1626	0.79 (0.46, 1.34)	n = 149	n = 299	0.58 (0.24, 1.39)
≥ One Dose Vaccine	153	1490		137	283	
Unvaccinated	18	136		12	16	
**Any Dose vs None NT (Rotarix)**	n = 83	n = 753	1.10 (0.50, 2.41)	n = 71	n = 123	1.40 (0.34, 5.80)
≥ One Dose Vaccine	75	676		68	114	
Unvaccinated	8	77		3	9	
**Any Dose vs None WA (RotaTeq)**	n = 88	n = 873	0.56 (0.27, 1.16)	n = 78	n = 176	0.30 (0.09, 0.98)
≥ One Dose Vaccine	78	814		69	169	
Unvaccinated	10	59		9	7	
**Any Dose vs None < 12 mths**	n = 48	n = 449	0.48 (0.22, 1.02)	n = 26	n = 37	0.28 (0.03, 2.83)
≥ One Dose Vaccine	37	392		21	36	
Unvaccinated	11	57		5	1	
**Any Dose vs None ≥ 12mths**	n = 134	n = 1177	1.22 (0.55, 2.73)	n = 123	n = 262	0.81 (0.29, 2.28)
≥ One Dose Vaccine	116	1098		116	247	
Unvaccinated	7	79		7	15	
**Full Dose vs None**	n = 129	n = 1008	0.83 (0.43, 1.58)	n = 121	n = 264	0.55 (0.23, 1.32)
Fully Vaccinated	111	913		109	248	
Unvaccinated	18	95		12	16	
**Full Dose vs None NT (Rotarix)**	n = 68	n = 529	2.06 (0.62, 6.83)	n = 61	n = 117	1.27 (0.31, 5.23)
Fully Vaccinated	60	469		58	108	
Unvaccinated	8	60		3	9	
**Full Dose vs None WA (RotaTeq)**	n = 61	n = 479	0.40 (0.18, 0.93)	n = 60	n = 147	0.29 (0.09, 0.96)
Fully Vaccinated	51	444		51	140	
Unvaccinated	10	35		9	7	
**Full Dose vs None < 12 mths**	n = 29	n = 194	0.45 (0.15, 1.39)	n = 21	n = 35	0.23 (0.02, 2.43)
Fully Vaccinated	18	161		16	34	
Unvaccinated	11	33		5	1	
**Full Dose vs None ≥ 12mths**	n = 100	n = 814	1.08 (0.47, 2.44)	n = 100	n = 229	0.79 (0.28, 2.22)
Fully Vaccinated	93	752		93	214	
Unvaccinated	7	62		7	15	
**Full Dose vs Partial Dose**	n = 153	n = 1350	0.65 (0.42, 1.01)	n = 137	n = 283	0.63 (0.35, 1.13)
Fully Vaccinated	111	1060		109	248	
Partially Vaccinated	42	290		28	35	
**Full Dose vs Partial Dose NT (Rotarix)**	n = 75	n = 620	0.58 (0.29, 1.14)	n = 68	n = 114	0.33 (0.11, 0.99)
Fully Vaccinated	60	535		58	108	
Partially Vaccinated	15	85		10	6	
**Full Dose vs Partial Dose WA (RotaTeq)**	n = 78	n = 730	0.71 (0.40, 1.26)	n = 69	n = 169	0.83 (0.40, 1.71)
Fully Vaccinated	51	525		51	140	
Partially Vaccinated	27	205		18	29	
**Full Dose vs Partial Dose < 12 mths**	n = 37	n = 314	0.49 (0.19, 1.28)	n = 21	n = 36	0.23 (0.03, 1.62)
Fully Vaccinated	18	183		16	34	
Partially Vaccinated	19	131		5	2	
**Full Dose vs Partial Dose ≥ 12mths**	n = 116	n = 1036	0.70 (0.42, 1.16)	n = 116	n = 247	0.78 (0.41, 1.50)
Fully Vaccinated	93	877		93	214	
Partially Vaccinated	23	159		23	33	

pathogens

MDPI

Table 3. Odds Ratio of vaccination in rotavirus cases versus controls in additional population-based analysis i) children age-eligible for full vaccination only (aged ≥ 24 weeks in the NT and ≥ 32 weeks in WA), ii) children aged < 5 years only, iii) Aboriginal children only, and iv) 'missing dose assumption' removed.

	NT > 24wks & WA > 32 wks			< 5 years			Aboriginal Children Only			No Missing Dose Assumption		
**Immunisation Status**	**Cases**	**Controls**	**Odds Ratio (95% CI)**	**Cases**	**Controls**	**Odds Ratio (95% CI)**	**Cases**	**Controls**	**Odds Ratio (95% CI)**	**Cases**	**Controls**	**Odds Ratio (95% CI)**
**Any Dose vs None**	n = 149	n = 1416	0.91 (0.49, 1.71)	n = 164	n = 1559	0.84 (0.48, 1.48)	n = 137	n = 1290	0.73 (0.41, 1.29)	n = 171	n = 1626	0.79 (0.46, 1.34)
≥ One Dose Vaccine	137	1313		148	1431		121	1178		153	1490	
Unvaccinated	12	103		16	128		16	112		18	136	
**Any Dose vs None NT (Rotarix)**	n = 71	n = 641	2.31 (0.71, 7.58)	n = 82	n = 745	1.06 (0.48, 2.34)	n = 71	n = 637	0.89 (0.40, 2.01)	n = 83	n = 753	1.10 (0.50, 2.41)
≥ One Dose Vaccine	68	582		74	670		63	573		75	676	
Unvaccinated	3	59		8	75		8	64		8	77	
**Any Dose vs None WA (RotaTeq)**	n = 78	n = 775	0.45 (0.21, 0.99)	n = 82	n = 814	0.64 (0.29, 1.43)	n = 66	n = 653	0.58 (0.26, 1.30)	n = 88	n = 873	0.56 (0.27, 1.16)
≥ One Dose Vaccine	69	731		74	761		58	605		78	814	
Unvaccinated	9	44		8	53		8	48		10	59	
**Any Dose vs None < 12 mths**	n = 26	n = 239	0.46 (0.16, 1.35)	n = 48	n = 449	0.48 (0.22, 1.02)	n = 43	n = 399	0.41 (0.18, 0.90)	n = 48	n = 449	0.48 (0.22, 1.02)
≥ One Dose Vaccine	21	215		37	392		32	348		37	392	
Unvaccinated	5	24		11	57		11	51		11	57	
**Any Dose vs None ≥ 12mths**	n = 123	n = 1177	1.22 (0.55, 2.73)	n = 116	n = 1110	1.56 (0.62, 3.98)	n = 94	n = 891	1.34 (0.52, 3.45)	n = 123	n = 1177	1.22 (0.55, 2.73)
≥ One Dose Vaccine	116	1098		111	1039		89	830		116	1098	
Unvaccinated	7	79		5	71		5	61		7	79	
**Full Dose vs None**	n = 121	n = 977	0.83 (0.44, 1.59)	n = 122	n = 951	0.91 (0.45, 1.83)	n = 103	n = 778	0.77 (0.38, 1.58)	n = 129	n = 993	0.84 (0.44, 1.60)
Fully Vaccinated	109	894		106	864		87	702		111	898	
Unvaccinated	12	83		16	87		16	76		18	95	
**Full Dose vs None NT (Rotarix)**	n = 61	n = 500	2.09 (0.63, 6.94)	n = 67	n = 521	1.97 (0.59, 6.55)	n = 59	n = 446	1.65 (0.49, 5.58)	n = 68	n = 519	2.10 (0.63, 6.95)
Fully Vaccinated	58	450		59	463		51	396		60	459	

Unvaccinated	3	50		8	58		8	50		8	60	
**Full Dose vs None WA (RotaTeq)**	n = 60	n = 477	0.40 (0.18, 0.93)	n = 55	n = 430	0.45 (0.18, 1.13)	n = 44	n = 332	0.39 (0.15, 1.00)	n = 61	n = 474	0.41 (0.18, 0.94)
Fully Vaccinated	51	444		47	401		36	306		51	439	
Unvaccinated	9	33		8	29		8	26		10	35	
**Full Dose vs None < 12 mths**	n = 21	n = 163	0.47 (0.15, 1.44)	n = 29	n = 194	0.45 (0.15, 1.39)	n = 26	n = 169	0.34 (0.10, 1.12)	29	190	0.47 (0.15, 1.44)
Fully Vaccinated	16	142		18	161		15	140		18	157	
Unvaccinated	5	21		11	33		11	29		11	33	
**Full Dose vs None ≥ 12mths**	n = 100	n = 814	1.08 (0.47, 2.44)	n = 93	n = 757	1.34 (0.52, 3.46)	n = 77	n = 609	1.17 (0.45, 3.07)	n = 100	n = 803	1.09 (0.48, 2.46)
Fully Vaccinated	93	752		88	703		72	562		93	741	
Unvaccinated	7	62		5	54		5	47		7	62	
**Full Dose vs Partial Dose**	n = 137	n = 1213	0.68 (0.43, 1.07)	n = 148	n = 1308	0.62 (0.40, 0.96)	n = 121	n = 1055	0.72 (0.44, 1.17)	n = 153	n = 1350	0.75 (0.48, 1.17)
Fully Vaccinated	109	1026		106	1026		87	810		111	1036	
Partially Vaccinated	28	187		42	282		34	245		42	314	
**Full Dose vs Partial Dose NT (Rotarix)**	n = 68	n = 558	0.62 (0.30, 1.28)	n = 74	n = 614	0.58 (0.29, 1.14)	n = 63	n = 517	0.73 (0.34, 1.56)	n = 75	n = 620	0.75 (0.38, 1.46)
Fully Vaccinated	58	504		59	529		51	438		60	519	
Partially Vaccinated	10	54		15	85		12	79		15	101	
**Full Dose vs Partial Dose WA (RotaTeq)**	n = 69	n = 655	0.72 (0.40, 1.29)	n = 74	n = 694	0.65 (0.36, 1.16)	n = 58	n = 538	0.70 (0.37, 1.35)	n = 78	n = 730	0.76 (0.42, 1.36)
Fully Vaccinated	51	522		47	497		36	372		51	517	
Partially Vaccinated	18	133		27	197		22	166		27	213	
**Full Dose vs Partial Dose < 12 mths**	n = 21	n = 177	0.57 (0.18. 1.78)	n = 37	n = 314	0.49 (0.19, 1.28)	n = 32	n = 270	0.40 (0.14, 1.11)	n = 37	n = 314	0.56 (0.22, 1.44)
Fully Vaccinated	16	149		18	183		15	160		18	178	
Partially Vaccinated	5	28		19	131		17	110		19	136	
**Full Dose vs Partial Dose ≥ 12mths**	n = 116	n = 1036		n = 111	n = 994	0.66 (0.40, 1.09)	n = 89	n = 785	0.85 (0.48, 1.51)	n = 116	n = 1036	0.81 (0.49, 1.34)
Fully Vaccinated	93	877		88	843		72	650		93	858	
Partially Vaccinated	23	159		23	151		17	135		23	178	
